# Focal dissection and rupture of left atherosclerotic subclavian artery: a rare cause of haemothorax

**DOI:** 10.1007/s12024-022-00529-7

**Published:** 2022-09-15

**Authors:** Rexson Tse, Melissa Thompson, Issac Han, Alex Olumbe

**Affiliations:** 1 Forensic and Scientific Services, Ground Floor, Health Support Queensland, Gold Coast University Hospital, Block EPathology 7 Education Building, 1 Hospital Boulevard, Southport, Queensland Australia; 2 Griffith University, School of Medicine and Dentistry, Southport, Queensland Australia; 3 Department of Molecular Medicine and Pathology, University of Auckland, Auckland, New Zealand

**Keywords:** Postmortem, Autopsy, Haemothorax, Atherosclerosis, Left subclavian artery, Rupture, Dissection

## Abstract

We report a rare case of a focally dissected and ruptured atherosclerotic left subclavian artery leading to haemothorax. A man in his 50 s who suffered from hypertension and gout was found dead in bed unexpectedly. Postmortem examination showed a focally dissected and ruptured atherosclerotic left subclavian artery with relatively disease-free aorta and major branches. Although theoretically possible, focal atherosclerosis of left subclavian artery compounded by hypertension causing focal dissection and rupture is not previously reported in literature.

## Case report

This case was a man in his early 50 s who was found dead at home unexpectedly. He suffered from gout and hypertension which was medically treated. He was last contacted on the morning of his death by one of his family members in which no concerns were raised. In the afternoon, he was found lifeless in bed in his residence. He had no recent history of trauma or surgical procedure (especially to the neck), or complaints of dizziness or light-headedness leading up to his death. No suspicious circumstances were raised.

**Fig. 1:**
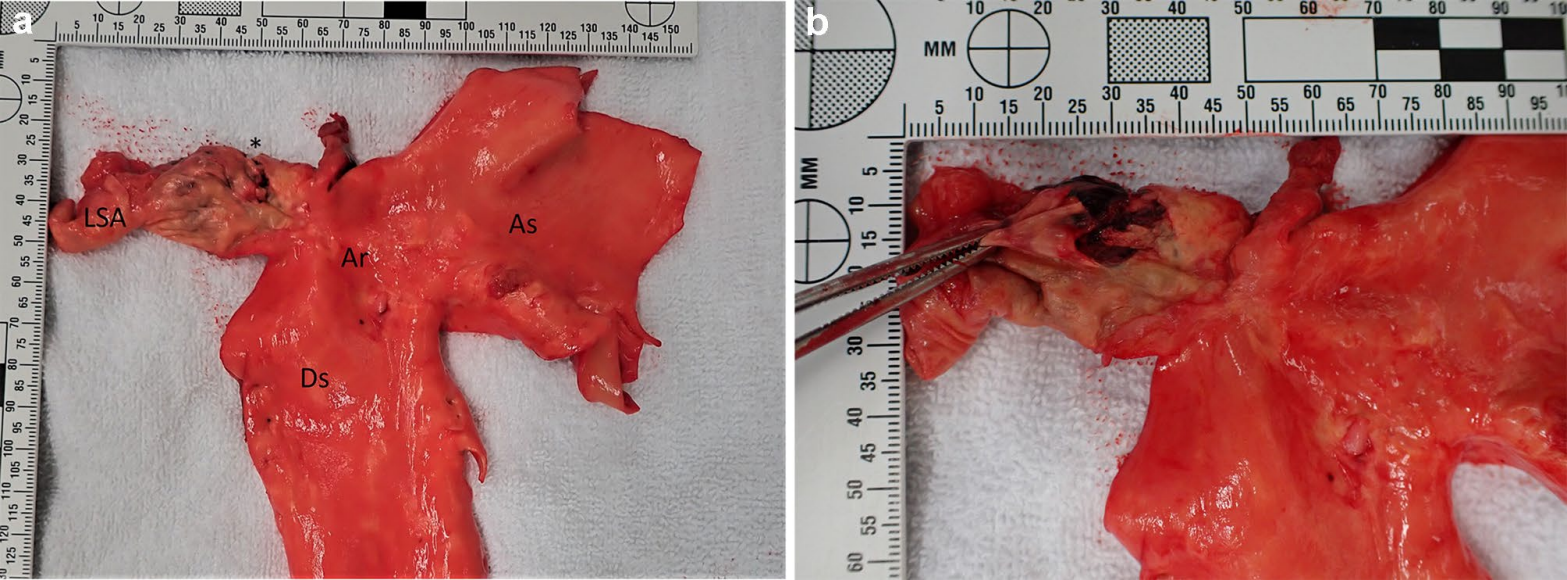
**A** Macroscopic photo of the ascending (As), arc (Ar) and descending thoracic (Ds) aorta. The left subclavian artery (LSA) was atherosclerotic and slightly aneurysmal with a site of focal dissection and rupture (*) in the proximal segment. Note the lack of atherosclerosis in the aorta and other branches. **B** A close-up image of the site of focal dissection and rupture

**Fig. 2:**
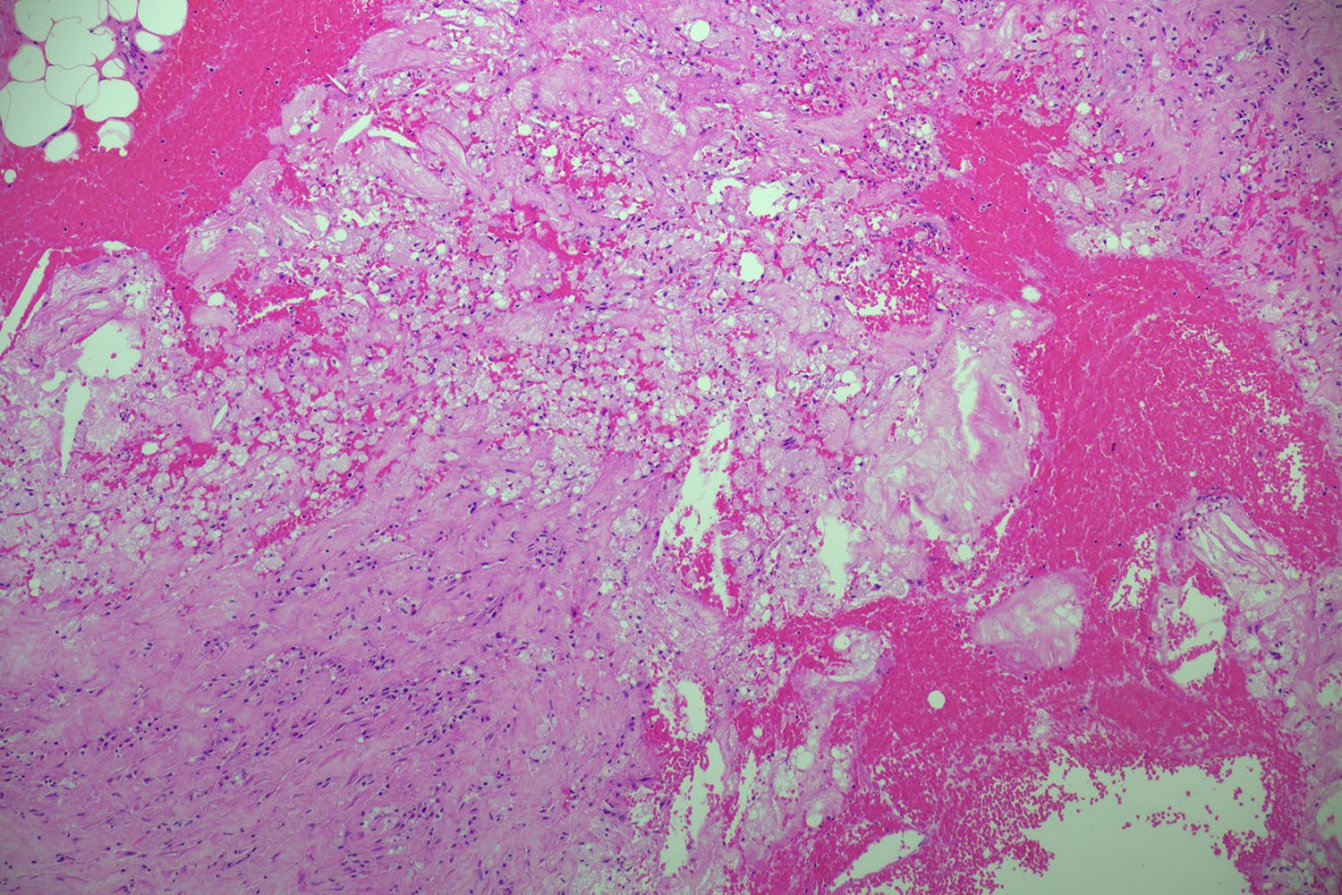
Microscopic image of the left subclavian artery showing atherosclerosis, focal dissection and rupture. No underlying connective tissue disorder or evidence of dissection

Postmortem examination was performed a day after the death. The deceased had a body mass index of 34.0 kg/m^2^ (height 170 cm, weight 108 kg) with truncal obesity; otherwise, the external examination was unremarkable with no disproportion of limb development or injuries on the body. The most striking finding at internal examination was in the chest cavity. The pleural cavity had 3 L of left-sided haemothorax. This originated from a focally dissected and ruptured left subclavian artery (Fig. 1). The rupture site was 10 mm distal to the orifice and had surrounding atherosclerosis. The artery itself arose normally and the orifice was widely patent. Microscopic examination confirmed the macroscopic finding and had no evidence of primary vasculitis or inherited connective tissue disorder (Fig. 2). The aorta, other branches of the aorta, pulmonary arteries and thoracic venous system were intact with no other site of rupture or identifiable bleeding source. Of interest, the ascending, arc and thoracic descending aorta had normal calibre with normal anatomy and distribution and only minimal focal atheroma. The heart was enlarged (550 g) with minimal coronary artery atherosclerosis, in keeping with a history of hypertension. The remaining visceral organs did not have any significant pathologies and no evidence of localised trauma.

The cause of death in this man was left haemothorax, secondary to a focally dissected and ruptured atherosclerotic left subclavian artery. Hypertension was thought to be a contributing factor to the death.

## Discussion

We report a rare case of spontaneous focal dissection and rupture of an atherosclerotic left subclavian artery causing haemothorax. A non-traumatic dissection and rupture at this location causing haemothorax is rare. Most haemothoraces are traumatic in nature with only a small number of natural causes [1]. Natural causes of haemothorax are frequently due to acute rupture of major blood vessels, coagulopathy, rupture of lung adhesions and inflammatory conditions, or chronically from connective tissue disorder or malignant processes [1, 2]. The source of haemothorax is frequently from a ruptured dissecting or aneurysmal aorta and rarely from other vessels [2].

Atherosclerosis is by far the most common pathology reported in the left subclavian artery, but other relatively rarer pathologies including arteritis, inflammation due to radiation exposure, compression syndromes, fibromuscular dysplasia and neurofibromatosis have been reported [3, 4]. In these conditions, there are only a small number of case reports reporting left subclavian rupture and are exclusively from underlying connective tissue disorder or vascular anomalies of the aortic arch [5–7]. Other causes of rupture at this site include trauma or catheterisation [7]. Although theoretically possible, atherosclerotic blood vessels with underlying hypertension can result in focal dissection and subsequent rupture of any artery. However, to the best of our knowledge there is no case reported on the left subclavian artery in forensic literature [2, 6]. Of peculiar interest is that the focal distribution of the atherosclerosis isolating in the left subclavian artery with the aorta and other branches is almost unaffected. In this case, it is unclear why atherosclerosis was localised in the left subclavian artery and not the aorta nor in the right side. This may be related to higher turbulent and/or pulsatile blood flow into the left subclavian artery as previously hypothesised [8]. This is evident in the atherosclerosis being proximal and near the orifice. This observation is also in keeping with the heterogenicity of atherosclerotic distribution in blood vessels observed in clinical literature [9, 10]. Although rare, in non-traumatic causes of acute haemothorax where an aortic source is not evident, exploring the branches of the aorta (such as the left subclavian arteries, as presented in this case) and other major vessels may identify rare causes of haemothorax.
